# Cats Did Not Change Their Problem-Solving Behaviours after Human Demonstrations

**DOI:** 10.3390/ani13060984

**Published:** 2023-03-08

**Authors:** Minori Arahori, Ayano Kimura, Saho Takagi, Hitomi Chijiiwa, Kazuo Fujita, Hika Kuroshima

**Affiliations:** 1 Wildlife Research Center, Kyoto University, Kyoto 606-8203, Japan; 2 Research and Development Section, Anicom Speciality Medical Institute Inc., Yokohama 231-0033, Japan; 3 Department of Psychology, Graduate School of Letters, Kyoto University, Kyoto 606-8501, Japan; 4 Japan Society for the Promotion of Science, Tokyo 102-0083, Japan; 5 Department of Animal Science and Biotechnology, Azabu University, Yokohama 252-5201, Japan

**Keywords:** cats, *Felis catus*, inhibitory control, social learning

## Abstract

**Simple Summary:**

Dogs can learn how to solve problems by watching humans. However, whether this is also true for cats, which are also companion animals, is unknown. In this study, three experiments were used to investigate whether cats could change their behaviour and gain rewards efficiently by observing a human demonstrating how to obtain food. We found no evidence that observing human behaviour enabled more efficient problem-solving by cats or caused them to change their behaviours. Other than their learning ability, the biological characteristics of cats and the experimental context may have contributed towards the present negative results.

**Abstract:**

Humans learn by observing the behaviour of others, which can lead to more efficient problem-solving than by trial-and-error learning. Numerous studies have shown that animals, other than humans, are also capable of social learning. Dogs, as humans’ closest companion animals, can learn to obtain rewards following behavioural demonstrations by humans. However, it is not known whether cats, who also live with humans, can learn how to solve problems by observing human behaviours. Three experiments were used to investigate whether cats could change their behaviour and gain rewards efficiently by observing a human demonstrating how to obtain food. In Experiment 1, a human demonstrated how to open a transparent drawer and take out the reward inside, but cats did not significantly follow the same method as the human. In Experiment 2a, a transparent tube device was used to make the operation easier for cats. However, cats were not influenced by the human behaviour. As the devices used in these experiments were transparent, meaning that the cats could see the food inside directly, they might have required strong inhibitory control. Therefore, in Experiment 2b the tube device was made opaque, and cats again observed the human demonstration. Nevertheless, the cats were not influenced by the human’s behaviour. The results of these experiments indicate a lack of social learning, including imitation, from human behaviours in cats, at least in these experimental settings with food rewards. Other than their inherent ability, cats’ biological characteristics and the experimental context may have contributed towards the negative results.

## 1. Introduction

Observing others and learning how they solve problems is often more efficient than individual trial-and-error learning. By learning novel behaviours from experienced individuals (sometimes through imitation), we improve our adaptation to our environment and accumulate solutions as traditions. Many studies have shown that non-human animals also have the capacity for social learning [1]. Whiten and Ham [2] define social learning as the behaviour of one individual influencing the behaviour of another individual, and at least part of that behaviour being learnt. Social learning thus includes being attracted to a specific stimulus or location by another individual (local enhancement), copying at least part of the behaviour of another individual (imitation) and reproducing the same outcome by whatever means (goal emulation) [2]. Examples of social learning in animals, including copying behaviour and efficient solving of problems, are now being revealed in laboratory experiments and field studies. However, possible alternative explanations are debated (see [3] for a review).

Learning how to solve problems not only from other conspecifics but also from heterospecifics sharing the same environment may be advantageous. In dogs (*Canis familiaris*), which are human companion animals with a long history of living closely with humans, various studies have explored social learning from humans. For example, Pongrácz et al. [4] placed dogs’ favourite toys or treats as a reward behind a V-shaped fence and measured the latency of the dogs to detour the fence to obtain the reward. The latency to obtain the reward did not shorten much when dogs had to detour the fence on the outside, even after six trials, without seeing any demonstration. In contrast, latency was shortened after a few trials when a human (the owner or the experimenter) demonstrated how to detour the fence. In another study, dogs quickly learnt to manipulate the handle attached to a box when they saw their owners demonstrate, which is a case of local enhancement [5]. In addition to that, dogs selected empty containers more often when a human demonstrator selected an empty container instead of a container with food [6]. It may be argued that dogs prioritise human communication cues.

Furthermore, dogs can imitate human behaviours. Using the ‘Do as I Do’ paradigm, dogs were trained to map various human movements into their motor schema, based solely on observation, and imitate them without the use of rewards [7]. However, dogs do not always copy human movements; they exhibit ‘goal emulation’ when the purpose of the action is clear, and ‘copying’ when the purpose is unclear [8]. Dogs have also been observed to make the same choices as other dogs and humans for the same outcome, for example, opening a door towards the left or the right [9]. It seems clear that human demonstrations can strongly influence dogs, at least in some contexts. It is possible that dogs’ social learning ability, which is present even in eight-week-old puppies [10], is related to their ancient domestication history and the strong selection pressure for tameness and suitability for human purposes [11]. Other domesticated animals such as horses (*Equus caballus*) [12] and goats (*Capra hircus*) [13] have also been shown to exhibit social learning from humans in detour tasks, suggesting that several species that have lived closely with humans are sensitive to human signals and behaviour as cues for solving problems.

Cats (*Felis catus*) were domesticated by humans about 9,000 years ago [14] and, like dogs, are close companion animals. In the social cognitive domain, recent studies have shown that cats can understand pointing [15], show social referencing (looking at their owner’s face for information in the presence of frightening or unfamiliar situations) [16] and discriminate human facial expressions [17]. Additionally, cats recognise their owners’ voice [18] and form cross-modal representations of the face and voice of their owners [19].

Concerning social learning in cats, the role of conspecific demonstrators has been investigated. Herbert and Harsh [20] examined behavioural changes in five different problem-solving situations with food after cats observed the solutions enacted by trained conspecific models. They reported that if a task was relatively easy and within the normal abilities of cats (e.g., moving a cart, pulling a string), observation of the model resulted in shorter latencies to solve the task. They concluded that more natural situations for cats were more likely to be solved successfully. John et al. [21] found that cats learnt an avoidance response when they heard a buzzer stimulus, and a food acquisition response from observing another cat. However, to our knowledge, social learning from human demonstrations has not yet been examined in cats, nor has their ability to imitate human behaviour as described above in dogs [9]. 

In this study, three experiments were conducted to investigate whether cats can learn to solve problems by observing human behaviour. In Experiment 1, we used a ‘drawer’ device. Cats had to scratch a tray on the device to obtain a reward. This scratching behaviour occurs naturally in the behavioural repertoire of cats [22], and pet cats are often given clawing toys. Therefore, to solve the first problem the cat had to place their paws on the tray and pull it towards their body, in what we considered to be an ecologically valid task. Cats were divided into two groups. After a baseline trial with no demonstration for both groups, cats in one group were given a demonstration by the experimenter (Demo group), and cats in the other group were not (No-demo group). We compared the number of rewards obtained and which parts of the device were touched. We hypothesised that if cats showed social learning through local enhancement or imitation, the following predictions would hold true: (1) cats in the Demo group would obtain more rewards than cats in the No-demo group; (2) cats in the Demo group would show increased touching of the site touched by humans compared to the baseline, while touches to other sites would either decrease or remain unchanged. The behaviour of cats in the No-demo group should not change.

In Experiment 2, we investigated whether cats would solve a problem more efficiently after a human demonstration and whether their choices were influenced even in the absence of effects on efficiency, as in Kis et al. [23]. In their study, bearded dragons were shown a video of a conspecific opening a sliding door. Those that saw the video showed a higher frequency of the same problem-solving behaviour as in the video, including the direction in which the door was slid. We used an apparatus similar to the cylinder task used in MacLean et al.’s [24] study of inhibitory control in several species. To obtain the reward, which was inside a transparent tube, cats first had to move away from the reward and go to one of the ends of the tube. We again compared experimental (Demo) and control (No-demo) groups on the number of rewards obtained and the latency to obtain the reward. Additionally, as the tube was open at each end, we asked whether the cats were influenced by the direction from which the human inserted a hand to retrieve the reward or whether the human used a hand or their head to retrieve the reward. We hypothesized that if cats showed social learning, i.e., performed more efficiently after observing the human demonstration, then compared to cats in the No-demo group, cats in the Demo group would (1) detour the transparent wall more often, (2) obtain the reward faster, and (3) obtain more rewards overall. In addition, if imitation or local enhancement occurred even when unnecessary, then cats in the Demo group would (4) tend to retrieve the reward from the same tube entrance as the human and (5) use their paws (the human used a hand) rather than their head to retrieve the reward.

Finally, in a supplementary experiment, we replaced the transparent tube with an opaque one to eliminate the inhibitory control aspect of the task. Thus, the only purpose of this experiment was to determine whether cats preferred the entrance of the tube used by the human when retrieving the reward. We hypothesised that if local enhancement influenced the cats’ choice, then they should select the entrance into which the human demonstrator inserted a hand tube to retrieve the reward.

## 2. Materials and Methods

### 2.1. Experiment 1: Drawer Task

#### 2.1.1. Participants

In this and subsequent experiments, we recruited cat owners who volunteered to participate via personal communication and our research group’s website. Forty-five cats at least 4 months old participated. Fifteen of them were excluded because of low motivation, failure to complete all trials, or persistent lack of interest in the device (12), or because of procedural errors (3). Therefore, data from 30 cats (21 males, 9 females) were analysed (see Table S1 for details). Their estimated mean age was 4.2 years (SD: 3.8; range: eight months–13 years). However, the age of the three cats was unknown. Twenty-two cats were mixed breed and likely originally feral; the remaining eight were purebred. Twenty-four cats lived in cat cafés, four were household cats, and two lived at the laboratory in the university. Cat cafés in Japan usually keep up to around 10 cats, and clients can pet and play with them. Cats kept in the laboratory could move freely around their rooms in a group during the day and stayed in individual cages at night. No cats were food or water deprived for the experiment. The cats were randomly divided into the Demo and No-demo groups, each containing 15 cats.

#### 2.1.2. Apparatus

**Figure 1:**
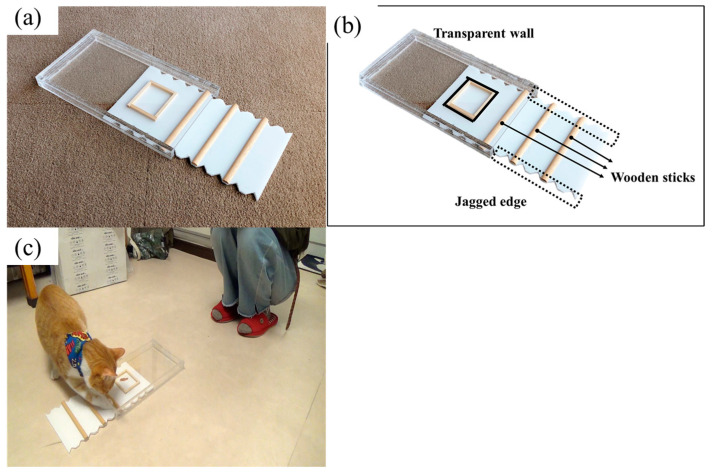
The drawer task apparatus (Experiment 1). (**a**) The apparatus in the “open” position; (**b**) Names of three parts of the apparatus; (**c**) An example of a trial.

The ‘Drawer’ was made from a transparent acrylic box (Width 18 cm × Height 2.5 cm × Length 32 cm) with an opening on one side and a white plastic tray (31 cm × 16 cm), which covered the floor inside the box. Three half-cylindrical wooden sticks were fixed parallel on the tray, at the far end of which a 6.5 cm × 5 cm area demarcated by a wooden frame was where the reward was placed (see Figure 1a: open, and Figure 1b: location and name of each part of the drawer apparatus). To solve the task, the cat had to pull the tray out by scratching at the wooden sticks on the trays with their paws, or at the jagged edges on the sides of the tray (Figure 1c). In this and subsequent experiments, the food (high-value and favourite treats for cats according to the owners) used as the reward was provided by the owners.

#### 2.1.3. Procedure

The experiment was conducted in a familiar room where each cat lived. This and the other experiments received the approval of the Animal Experiments Committee of the Graduate School of Letters, Kyoto University (21–3), and consent of the owners. The owners were also informed that they could stop the experiments at any time. 

We first confirmed that cats would eat food near the experimenters. The demonstrator (female) presented the cat with only the white plastic tray on which a reward had been placed (placing the reward was done out of sight). The holder (female) gently held the cat approximately 60 cm away from the tray (or closer, if necessary, to enable the cat to see the tray easily). The demonstrator sat behind the tray and used her fingers and voice to draw the cat’s attention to the reward, after which the holder released the cat. If the cat took the reward, we proceeded to the main experiment. A trial was stopped after approximately 30 s if the experimenters agreed that the cat was trying to leave, was not interested in the reward, or appeared afraid. Except for the demonstrator attracting the cat’s attention, during each trial, the demonstrator and the holder avoided eye gaze with the cat by looking only down at the floor. This was also the case in subsequent experiments.

The experiment consisted of three trials per cat. On the first trial, there was no demonstration for either group. The holder gently held the cat approximately 60 cm from the apparatus. The demonstrator simply presented the apparatus, and when the cat appeared to have seen the reward on the tray, the holder released the cat to start the trial, which ended when the cat obtained the reward, or 30 s elapsed with no reward obtained (the second and third trials also ended this way).

The second and third trials were different in the Demo and No-demo groups and proceeded identically within each group. In the Demo group, the demonstrator sat next to the cat, facing the apparatus, and the cat observed the demonstrator scratching at the wooden sticks with both hands, thus pulling out the tray. The tray was then returned to the drawer, and the demonstration repeated until the cat gazed at it. After the final demonstration, the cat was allowed to eat the reward from the pulled-out tray. The apparatus was then baited again out of the cat’s view, and the drawer was presented as in the first trial.

In the No-demo group, the cat was given the reward from the pulled-out tray one time, without any scratching behaviour by the demonstrator, who sat on the opposite side of the apparatus from the cat. The reward was presented after it had been placed in the drawer, as in the first trial. In other words, the groups differed only in observing scratching behaviour by the demonstrator or not. 

The experiment and those that followed were recorded using HDR-CX390 (SONY, Tokyo, Japan) and GZ-RX670-A (JVCKENWOOD, Kanagawa, Japan) video cameras and a GoPro HERO 4 (Woodman Labs, San Mateo, CA, USA) to capture the behaviour of the cats. 

#### 2.1.4. Analysis

First, the number of cats that obtained the reward on their own within 30 s was compared between the Demo and No-demo groups. Next, we examined whether there was more scratching at the wooden sticks in the Demo group than in the No-demo group. We also analysed whether the occurrence of attempts to obtain the reward directly through the transparent wall or by scratching at the jagged edges differed between the two groups, as these behaviours were never presented in a demonstration. Problem-solving attempts were defined as touching and moving paws to pull out the tray (simply touching or smelling the tray was not sufficient). We rated whether responses occurred (1) or not (0) for each part of the apparatus. We did not consider latency or duration due to the difficulty of identifying precise onset and ending. Trial 1 was regarded as the baseline, and changes in behaviour directed at each part were calculated for each individual by subtracting the score (0 or 1) of trial 2 from trial 1 and of trial 3 from trial 1. The value ‘−1′ meant the behaviour occurred in trial 1 but not in trials 2 or 3; ‘0′ meant no occurrence in either trial, or occurrence in both trials; and ‘1′ meant no occurrence in trial 1 but occurrence in at least trial 2 or 3. 

The videos were analysed frame by frame (30 frames/s). Adobe Premiere Pro CC v22.1.2 (Adobe Inc., San Jose, CA, USA) was used for the analysis in this and subsequent experiments. Videos were rated by M.A and by a second rater, naïve to the aims of the experiment. In the event of disagreement, a decision was made by consensus, all ratings were agreed upon by the two raters. The ‘mannwhitneyu’ command of the stats library [25] for Mann-Whitney U was used in Python 3.10.1 to test for statistical significance of differences between the groups.

### 2.2. Experiment 2a: Transparent Tube Task

#### 2.2.1. Participants

Forty-nine cats aged at least 4 months old participated. Seven cats were excluded from the analysis due to a lack of motivation during the main experiment (no contact with the tube or failure to complete all trials), and two were excluded due to experimental error. Data from a total of 40 cats (23 males, 17 female) were analysed. Details of the cats are shown in Table S2. The mean age was 3.5 years (SD: 2.3, range: 1–10 years and 2 months). Note that the ages of mixed-breed cats may be estimates for the same reason as in Experiment 1. Twenty-seven cats were mixed breeds and 13 were purebreds. Thirty-three cats lived in cat cafés, while the others were household cats. Twenty cats were randomly assigned to the Demo group, and 20 were assigned to the No-demo group.

#### 2.2.2. Apparatus

**Figure 2:**
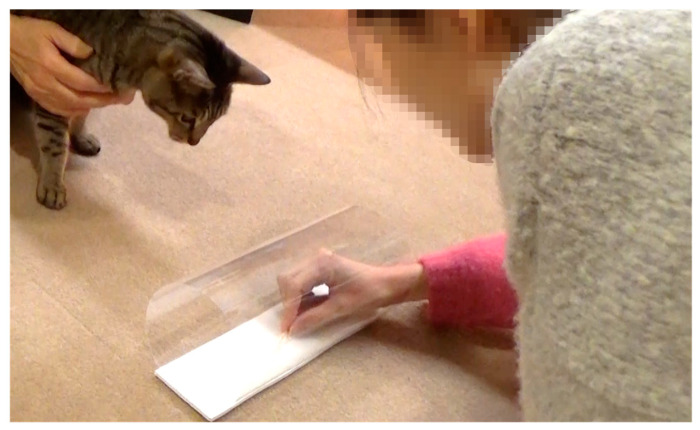
Set-up of the transparent tube task (Experiment 2a).

The apparatus consisted of a transparent plastic tube (11 cm in diameter, 26 cm long), open at both ends. It was large enough for a human to insert a human hand and a cat to insert its head. The tube was stuck to a plastic board base which was fixed horizontally on the floor with double-sided tape (Figure 2). A 450 × 300 × 10 mm black screen was used to hide the baiting procedure from the cat.

#### 2.2.3. Procedure

The tube was placed in front of the cat, between it and the demonstrator (Figure 2). The holder gently restrained the cat approximately 60 cm away from the tube. In the Demo group, an opaque screen was placed between the cat and the apparatus, and then the demonstrator inserted the reward, placing it in the middle of the tube. Next, the screen was removed, the demonstrator got the cat’s attention by calling and making eye contact, and then demonstrated how to obtain the reward from the apparatus by inserting one hand, withdrawing the reward and offering it to the cat. The demonstrator returned the screen to in front of the tube and inserted a reward into the tube. The demonstrator again removed the screen and drew the cat’s attention to the reward by pointing, eye contact, and talking. Once the experimenters were sure that the cat had seen the reward, the holder released it, and the trial began. The demonstration lasted approximately 6 s.

For the No-demo group, the screen was in place, and the demonstrator held the reward with both hands behind it. She reached forward and allowed the cat to take the reward from her hands. The procedure was otherwise the same as for Demo group.

The trial ended when the cat obtained the reward, or 30 s had elapsed. For the Demo group, the experimenter placed her hand into the tube from either the left (10 cats) or right (10 cats) entrance. This direction was the same throughout the five trials for each cat.

#### 2.2.4. Analysis

The actions of the cats towards the tube on each trial were categorised as follows: touching the transparent wall, choosing the right or left entrance (i.e., the same as or different from the human hand) without touching the wall, and none of the above (First action). When the cats finally obtained the reward or did not (last action), actions of the cats were categorised as follows: choosing the right or left entrance and none of the above. Additionally, included in the analysis was whether the cat attempted to obtain the reward with its head or paws; in the case of both, the first action was recorded.

The analyses aimed to clarify: (1) whether the two groups would differ in the number of trials with attempts to obtain the reward without detouring; (2) whether the latency to obtain the reward differed between the groups; (3) whether the total number of rewards obtained differed between the groups; (4) whether cats in the Demo group were influenced by the direction in which the human inserted her hand into the tube on the first rewarded trial (only the first trial was included to rule out learning across trials); and (5) whether cats in the Demo group were more likely to use their paws than those in the No-demo group.

The video coding was conducted by M. A. Four videos, i.e., 20% of the videos in each group were analysed by a 2nd coder who was naïve to this experiment. For both the Demo and the No-demo group, the agreement for measures other than latency was 100%. Statistical analysis was performed in Python 3.10.1 using the ‘intraclass_corr’ command [26] of the Pingouin library to assess inter-rater reliability for latency. The intraclass correlation of average fixed raters in latency using was 0.99, meaning the agreement was sufficient.

The ‘mannwhitneyu’ command for Mann–Whitney U in the stats library was used to look at differences between groups in analysis (1) [25]. For analyses (2), (3) and (5), the lme4 package [27] and the car packages [28] from R 4.1.3 [29] were used. In analysis (2), the least squares method was used for parameter estimation in the linear mixed model (LMM) and a type III test was performed. In analyses (3) and (5), the binomial distribution (link = “logit”) was assumed in the generalised linear mixed model (GLMM), the maximum likelihood method was used for parameter estimation, and a type III test was performed. In analysis (4), a binomial test was performed.

### 2.3. Experimant 2b: Opaque Tube Task

#### 2.3.1. Participants

Thirty cats aged at least 4 months participated. Five were excluded due to a lack of motivation because cats who obtained a reward on at least one trial were included and completed all trials. Furthermore, two cats were excluded due to fear of the tube, two due to experimenter errors, and one due to corruption of the experimental data. In total, data from 20 cats (males = 15, females = 5) were analysed. Details of the participant cats are presented in Table S3. The mean age was 4.5 years (SD: 3.2, range: 6 months–13 years). Of these, 16 were mixed breed and four were purebreds. Note the age estimation of mixed breeds for the same reason as in Experiment 1. Sixteen cats lived in cat cafés, one was household cats, and three lived in the university. Three had participated in Experiment 2a, approximately two years earlier.

#### 2.3.2. Apparatus

The apparatus was the same as in Experiment 2a, except it was made of white (opaque) plastic.

#### 2.3.3. Procedure

The basic procedure was the same as in Experiment 2a. The experimenter presented both entrances of the tube to the cat, thus showing the reward inside the tube. She then gave the reward to the cat, and then baited the tube with a new reward. The left-right order in which the entrances shown was consistent within and counterbalanced between individual cats.

#### 2.3.4. Analysis

Coding (conducted by M. A.) focused on whether cats used the same tube entrance as the human demonstrator to retrieve the reward. Four videos (20% of the total) were analysed by a 2nd coder who was naïve to the aim of the experiment. The inter-coder agreement was 100%. For analysis, a binomial test was planned to be used to determine whether first rewarded trials were solved using the same tube entrance as the human. 

## 3. Results and Discussions

### 3.1. Experiment 1: Drawer Task

#### 3.1.1. The Number of Cats That Obtained the Reward

Two cats in the Demo group and one in the No-demo group obtained the reward in trial 1 and were excluded from the analysis. Only two cats in each group solved the task in trials 2 or 3. As there was no apparent difference between the groups, no statistical analysis was performed. Therefore, contrary to our expectation, cats showed no evidence of solving the problem more efficiently after seeing the human demonstration. 

#### 3.1.2. Changes in Working on Each Part

**Figure 3:**
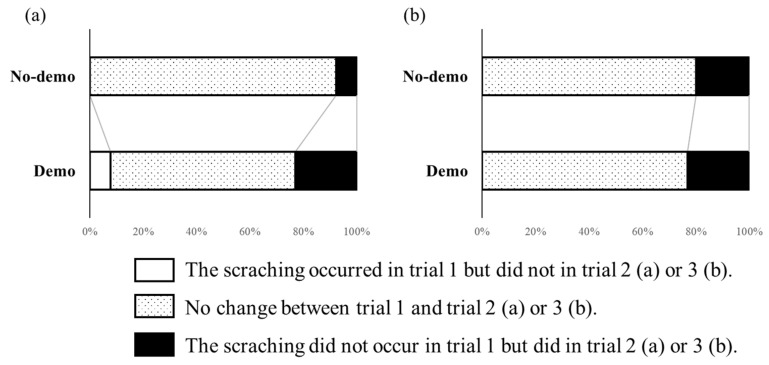
Changes between trial 1 and trial 2 (**a**) and trial 3 (**b**) in scratching the wooden sticks in experiment 1. White bar: scratching occurred in trial 1 but did not in trial 2 (**a**) or 3 (**b**); stippled bar: no change between trial 1 and trial 2 (**a**) or 3 (**b**); black bar: scratching did not occur in trial 1 but did in trial 2 (**a**) or 3 (**b**).

Differences between groups in manipulating the wooden sticks are shown in Figure 3. Changes from trial 1 to 2 and from trial 1 to 3 were not significantly different between the groups (Mann–Whitney *U* test; trial 1–2: *U* = 93.0, *p* = 0.92; trial 1–3: *U* = 92.5, *p* = 0.95).

The corresponding results for the other two parts are shown in Figures S1 and S2. The same analyses were performed for the transparent wall (Mann–Whitney *U* test; trial 1–2: *U* = 91.0, *p* = 0.97; trial 1-3: *U* = 121.0, *p* = 0.06) and jagged edge (Mann–Whitney *U* test; trial 1–2: *U* = 98.5, *p* = 0.68; trial 1–3: *U* = 81.0, *p* = 0.59); no differences were found between the groups.

One possible reason for the lack of evidence for social learning in this experiment is that the apparatus was too complex for the cats. Therefore, a simpler apparatus was used in Experiment 2.

### 3.2. Expeiment 2a: Transparent Tube Task

#### 3.2.1. First Actions with Touching the Transparent Wall

For the first action of each group, the number of attempts to touch the transparent wall to obtain the reward in the five trials was counted for each individual and averaged. The means were 2.20 (SD = 1.47) and 2.55 (SD = 1.36) times in the Demo and No-demo groups, respectively, with no significant difference (*U* = 180.5, *p* = 0.60).

#### 3.2.2. Latency to Obtain the Reward (Last Action)

This analysis concerned only trials where cats obtained the reward. The mean latency to obtain the reward was 396.3 frames and 380.4 frames for the Demo and No-demo groups, respectively. We set latency as the dependent variable and groups, trials, and their interaction as independent variables. No effects were revealed in the LMM analysis (groups: χ*2 (1)* = 0.33, *p* = 0.57; trials: χ*2 (1)* = 0.79, *p* = 0.37; groups × trials: *χ2 (1)* = 0.10, *p* = 0.75).

#### 3.2.3. Total Number of Rewards Obtained (Last Action)

The number of trials finally ending in the reward being obtained was compared between the groups. Cats in the Demo group obtained more rewards than those in the No-demo group on all trials, and more cats obtained the reward in the fifth trial than in the first trial (Table 1). 

For verification, for each trial, the dependent variable was assigned the value of 1 or 0 according to whether the cats obtained the reward or not, and we set groups, trials, and the interactions as independent variables, and participants as the random effect. However, no significant effects were found (group: *χ2 (1)* = 1.42, *p* = 0.23; trial: *χ2 (1)* = 1.90, *p* = 0.17; groups × trial: *χ2 (1)* = 0.06, *p* = 0.80).

#### 3.2.4. Did Cats Prefer the Tube Entrance into which the Experimenter Inserted Her Hand? (Demo Group, First Action, and Last Actions)

Only the first trials resulting in a reward were included in this analysis. Concerning the first action (i.e., excluding trials in which the cat first touched the transparent wall or made no choice), in 11 trials, cats retrieved the reward from the same entrance as the human, and in 7 trials, they retrieved it from the other entrance. A binomial test revealed no significant difference (probability = 61.1%, *p* = 0.48, 95% confidence interval [0.36, 0.83]). Concerning the final action, in 13 trials, cats got the reward from the same entrance as the human, compared to 7 trials from the other entrance, again a non-significant difference (probability = 65%, *p* = 0.26, 95% confidence interval [0.41, 0.85]).

#### 3.2.5. Body Parts Used to Obtain the Reward

Trials resulting in no reward were not included in this analysis. The Demo group had 16 trials by paws and 55 trials by the head; corresponding scores for the No-demo group were 13 and 43, respectively.

The dummy variable was changed to 1 for successful trials by paws and 0 for the head. This variable was used as the dependent variable. We set groups, trials, and the interaction as independent variables and participants as the random effect. There was no main effect of a group or a group x trial interaction according to the GLMM analysis. However, there was a significant effect of trial (trial: *χ2 (1)* = 5.22, *p* = 0.02, estimate = 2.74, standard error = 1.20; group: *χ2 (1)* = 1.41, *p* = 0.23; trial × group: *χ2 (1)* = 1.21, *p* = 0.27). The number of cats who used their paws increased with each trial to obtain the reward, regardless of group.

Based on these results, we could not conclude that the cats’ performance was altered by observing human demonstrations.

The number of trials in which cats used their paws increased with each trial, but there was no difference between the groups on any measure. In other words, learning to obtain the reward with their paws was a trial-and-error process rather than an effect of human demonstration. It may simply have been easier for some cats to use their paws because the width of the tube was about the same as a cat’s head, although most of cats still used their head.

A simple interpretation of these results was that cats did not show social learning towards humans. However, the cats showed several reward-directed behaviours without detouring the transparent wall, as also seen in another experiment (Arahori, in preparation). Even if cats are capable of social learning, limited inhibitory control in the presence of a visible reward might impair their ability to show it. Therefore, we conducted a supplementary experiment. In this experiment, we used an opaque tube to eliminate the cats’ tendency to respond directly to the reward without detouring. This allowed further investigation of whether cats were influenced by the direction of a human demonstrator’s hand movement.

### 3.3. Experiment 2b: Opaque Tube Task

#### Did Cats Use the Same Tube Entrance as the Human Demonstrator?

Only first trials in which cats were successful directly after the start were included. The successful trials involving the same entrance as the human demonstration to those involving the other entrance in each group were 10 and 10, respectively. Therefore, we did not conduct any statistical analysis. This result shows that the cats’ behaviour was not affected by the particular entrance of the opaque tube into which the human hand was inserted.

## 4. General Discussion

This study used three different apparatuses to assess social learning in cats observing human demonstrations of solutions to problems. We found no evidence of social learning in the experimental situations. In Experiment 1, cats that observed a human demonstrator solving a drawer task performed no better on the task than the control group that saw no demonstration; nor did they specifically touch the same parts of the apparatus as the human. In Experiment 2, a tube task was used, and again the demonstration did not reduce incorrect attempts by the cats or help them to solve the problem. The simplest conclusion to draw from these data is that cats failed to show social learning from humans in the context of food. Instead, cats tried to obtain the food by their own means. 

Relevant to our study is one in which, when faced with an unsolvable, food-related task, cats were less likely than dogs to refer to their owners [15]. It is conceivable that cats’ poor performance in social learning tasks is related to their domestication history, which differs considerably from that of dogs. African wildcats (*Felis silvestris lybica*), considered ancestral to modern cats, were solitary, which means fewer opportunities for social learning outside the parent-offspring relationship. Cats were domesticated later than dogs [11,14], and the origins of domestication were also different between dogs and cats. In dogs, qualities desired by humans, such as obedience and fidelity, have been artificially selected for contexts such as hunting and herding [11]. By contrast, artificial selection in cats has been much weaker; cats have fewer pre-adaptations for domestication and fewer practical roles [30]. Nonetheless, they are capable of learning from conspecifics [20,21]. Heyes [31] notes that attention and motivation are critical for successful social learning and appropriate social interactions. As examples of solitary species that excel at social learning, she cites the common octopus and the red-footed tortoise. Therefore, we need to consider other factors that might be related to social learning ability in cats.

Temperamental factors, including arousal level, may be relevant [32], along with the influence of stress on performance in test situations. As recommended [33], our experiments were conducted in locations familiar to the cats, but there were still novelties present, including the experimenters and equipment. These may have reduced the cats’ interest in the food, and, in fact, many cats stopped participating. As Delgado et al. [34] showed, cats prefer easily accessible rewards over rewards that require work, such as food puzzles. In all three experiments in the present study, the cats were rewarded during the demonstration to maintain motivation. However, it is possible that this procedure reduced their motivation. One implication is that tasks that do not involve food may be advantageous.

In dogs, vocal encouragement has been shown to facilitate social learning from humans [35]. In our study, an unfamiliar demonstrator tried to obtain the cat’s attention using voice and gestures, but this was not fully satisfactory. Possibly, a demonstration by the owner would be more attention-grabbing. However, while cats are known to be fearful of strangers [36], they do not always prefer their owners over strangers [37], and pet cats did not show cross-modal (face and voice) recognition of their owners [19]. Furthermore, ostensive cues given by an unknown human resulted in cats making fewer A-NOT-B errors more than cues given by their owner [38], suggesting that cats differ from dogs and human infants in their processing of human ostensive cues. Based on such studies, we think it unlikely that owners would make significantly better demonstrators than strangers in social learning tasks, at least in tasks such as those used here.

## 5. Conclusions

In the present study, human behavioural cues led to no social learning in cats. Their responses when presented with three tasks, which could lead to a food reward, were not influenced by human demonstrations. 

## Figures and Tables

**Table 1:** The proportion of cats finally obtaining the reward in each trial in experiment 2a.

Group	Trial 1	Trial 2	Trial 3	Trial 4	Trial 5
Demo	0.60	0.70	0.75	0.70	0.80
No-demo	0.35	0.60	0.65	0.55	0.65

## Data Availability

The data presented in this study are available on request from the corresponding author.

## Supplementary Materials

The following supporting information can be downloaded at: https://www.mdpi.com/article/10.3390/ani13060984/s1, Figure S1: Changes in working on the transparent wall; Figure S2: Changes in working on the jagged edge; Table S1: Individuals who participated in the experiment 1 (the drawer task); Table S2: Individuals who participated in the experiment 2a (the transparent tube task); Table S3: Individuals who participated in the experiment 2b (the opaque tube task).
